# Health-Related Messages About Herbs, Spices, and Other Botanicals Appearing in Print Issues and Websites of Legacy Media: Content Analysis and Evaluation

**DOI:** 10.2196/63281

**Published:** 2024-12-04

**Authors:** Ann Gaba, Richard Bennett

**Affiliations:** 1 Department of Environmental, Occupational, and Geospatial Health Sciences Graduate School of Public Health and Health Policy City University of New York New York, NY United States

**Keywords:** legacy media, health applications, health communication, botanical products, content analysis

## Abstract

**Background:**

Legacy media are publications that existed before the internet. Many of these have migrated to a web format, either replacing or in parallel to their print issues. Readers place an economic value on access to the information presented as they pay for subscriptions and place a higher degree of trust in their content. Much has been written about inaccurate and misleading health information in social media; however, the content and accuracy of information contained in legacy media has not been examined in detail. Discussion of herbs, spices, and other botanicals has been absent from this context.

**Objective:**

The objectives of this study were to (1) identify the health associations of botanical products mentioned in legacy media targeted to a range of demographic groups and (2) evaluate these health associations for accuracy against published scientific studies.

**Methods:**

In total, 10 popular magazines targeting a range of gender, race/ethnicity, and sexual orientation demographic groups were selected for analysis. Relevant content was extracted and coded over 1 year. Associations between specific botanical products and health factors were identified. For the most frequent botanical–health application associations, a PubMed search was conducted to identify reviews corresponding to each item’s indicated applications. Where no systematic reviews were available, single research studies were sought.

**Results:**

A total of 237 unique botanical products were identified. There were 128 mentions of these in the print issues and 1215 on the websites. In total, 18 health applications were identified and used to categorize the indicated uses for the various products individually and as general categories. The most frequently mentioned applications were skin care, with 913 mentions, immunity enhancement, with 705 mentions, gastrointestinal health and probiotics, with 184 mentions, and cognitive function (stress and mental health), with 106 mentions. Comparison to published literature evaluating the efficacy of these functions identified positive support for aloe vera, argan oil, chamomile, jojoba oil, lavender, rosemary, and tea tree oil in skin care. Berries, ginger, turmeric, and green tea had the strongest evidence for a role in immunity enhancement. Ginger and oats were supported as having a role in gastrointestinal health. Finally, berries, lavender, ashwagandha, and cannabidiol were supported as having a role in managing stress. Other frequently mentioned items such as aloe vera, ashwagandha, or mushrooms for immunity were less strongly supported.

**Conclusions:**

Comparison of the most prevalent associations between botanical products and health applications to published literature indicates that, overall, these associations were consistent with current scientific reports about the health applications of botanical products. While some products had a greater degree of research support than others, truly egregious falsehoods were absent. Therefore, legacy media may be considered a credible source of information to readers about these topics.

## Introduction

### Information Needs About Health Applications of Botanical Products

People from many different cultural backgrounds have used herbs, spices, and other botanical products to promote health and manage various illnesses. Gura et al [1] provide a review of some well-known examples. Some recent reviews in Western medicine have also identified these as being of value in immune support [2] and in the management of metabolic syndrome [3], type 2 diabetes [4,5], and osteoporosis [6].

The most recent application of these botanical products has been in the management of COVID-19 [7]. This literature has also drawn on the traditional practices of cultures with long histories of botanical medicine, such as those from Africa [8], China [9], and India [10]. Reporting based on peer-reviewed literature is crucial in providing appropriate guidance to both practitioners and consumers. Lenssen et al [11] caution against the widespread adoption of botanical medicines based on traditional uses alone.

Individuals needing information about the potential applications of botanicals may seek it on their own, with the internet being their primary source. Data-mining studies of searches on Google [12,13] and Twitter (subsequently rebranded X) [14] have identified strong trends toward seeking information about uses of botanicals and dietary supplements as prevention or treatment of COVID-19.

### Inaccurate Health Information Identified on Social Media

While searching the internet is a common way of obtaining health information [15], much has been written about inaccurate and misleading health information on the internet, especially through social media [16-18]. The vast proliferation of information from a wide range of sources with varying degrees of credibility has been termed an “infodemic” [19]. However, while these studies focused on social media and websites make important contributions to our understanding of health communications, they have largely excluded information from legacy media.

### Legacy Media as Part of the Media Landscape

Legacy media are publications such as newspapers and magazines that existed before the internet. While readership of these in print form has declined substantially in the past decades, some retain a loyal subscriber base, and many have migrated to the web while maintaining their print issues in some form [20]. Readers need to place an economic value on access to the information presented as they must pay for print subscriptions or access to web content. Conversely, editors of legacy media publications keep track of the characteristics of their subscriber base and select materials that will speak to their readers. Organization of content, length of articles, vocabulary, reading level, and tone are also part of the content production process. Failing to do so would alienate readers. This level of content curation differs from more open-ended content appearing in social media platforms, where there may be little or no limitations as to what information is posted.

Possibly related to the level of content curation, there is also evidence that there is a higher level of trust in the information provided by legacy media [21]. This is especially noteworthy among older readers and those from communities and specific identity groups where the most effective communications are those that are tailored to the needs, interests and vocabulary of each specific population [22-24]. In addition, more so than the general public, it is these target demographics who are most likely to have an interest in the application of botanical products to a range of health issues [25,26]. Therefore, it may be of interest to determine the extent to which information about botanicals found in legacy media is consistent with that found in published scientific studies.

### Comparing Print and Web Media

To maintain their readership levels and commercial viability, many popular magazines and newspapers have expanded their media footprints, producing both a print version and web version [20]. However, studies comparing health information within this hybrid matrix have been limited.

The textual formatting of the material itself can make a difference in how the information is read and processed [27]. This difference has been shown to affect educational efforts in public health [28-30]. It may also make a difference in people’s preferences in content that they seek for themselves, such as information about the health applications of botanical products.

### Need for Evaluation of Legacy Media Content About Botanical Products

The content and accuracy of the information contained in legacy media sources has not been examined in detail. Despite their importance to many population groups, discussion of herbs, spices, and other botanicals has been entirely absent from this context.

To better understand this aspect of health communication, this study examined what was presented to the public about the health applications of specific herbs, spices, and other botanical-derived products in a cross-section of popular magazines collected over 1 year. Through this content analysis, the most prevalent associations were identified and compared to published scientific literature. While the associations identified were not legally defined health claims or structure-function claims [31], the placement, wording, and accompanying illustrations nonetheless suggested that such relationships may exist. This can then be communicated to readers as prospective consumers via these media through both advertisements of products and discussions in articles. Making these comparisons will provide an evaluation of the degree of congruence of published research regarding the predominant health applications associated with identified botanicals for a range of health issues. Determining the degree of accuracy of these associations will make clear whether the trust of their readership is justified for this type of content. To achieve these goals, the aims of this study were to describe the content of legacy media discussing health applications of botanical products and compare this content with published scientific evidence.

## Methods

### Inclusion Criteria

A convenience sample of periodicals intended to be representative of a diverse cross-section of demographic and interest groups was used for this study. Magazines that were available at 2 high-traffic newsstands in New York City in December 2019 were screened for inclusion in the study. A total of 16 were purchased for further review. Of those 16 purchased magazines, 10 (62%) included one or more articles about a health-related theme, including herbs, spices, or other botanicals, and published both print and web issues throughout the year. These 10 popular magazines targeting a range of gender, race/ethnicity, and sexual orientation demographic groups were selected for inclusion in this study. These were *The Advocate*; *Elle*; *Essence*; *GQ*; *Men’s Journal*; *O, The* *Oprah* *Magazine*; *Out*; *Scientific American*; *Time*; and *Vogue*. Subscriptions were purchased for each of these, and relevant articles, advertisements, and editorials from both print and web issues were collected from January 2020 to December 2020.

### Ethical Considerations

All data were collected from publicly available periodicals through paid subscriptions by the first author. Because no human participants or animal subjects were included, the study was exempt from institutional review board review.

### Data Coding

Codes were developed in an iterative process as the coding was being conducted. One of the authors (RB) extracted the data from all discussions in articles, editorials, and advertising containing references to herbs, spices, or other botanicals. Unique identification codes for each botanical product were created as the data were extracted. New codes were added until no new items were identified. Coded data were entered into a spreadsheet during this process, with codes and product names listed separately in a code list. Components of multi-ingredient products were each coded separately. Following this, the other author (AG) used the created code list to recode the list of botanical products. Where discrepancies were noted, these were discussed to a point of consensus. Items that had been initially misidentified as botanicals were removed from the dataset.

Health applications of these products were identified in a similar manner. Codes were developed as the data were being extracted and reviewed for congruence. A total of 16 health applications were identified, with codes recorded for each. These were antimicrobial, blood sugar management, cognitive function, dental care, detoxifying, immunity enhancement, gastrointestinal health and probiotics, hair growth and repair, hormone regulation, energy increase, pain relief, cancer prevention, cardiovascular disease prevention, stress reduction, sexual enhancement, skin care, sleep aid, and weight loss.

Using the same process, 3 different methods of use were also identified: ingested, topical, and airborne. Ingested products consisted of food items and dietary supplements. Topical items were lotions, creams, or oils applied to the skin. Airborne products were to be inhaled in either a short-range aromatherapy or as whole-room scent enhancers.

Because there may be differences in readership between print and web issues and possibly different information exposure, all the information collected was identified as being derived from either the print or web issues of each periodical. This was done to compare content across media presentations and identify differences in the prevalence and presentation of information.

### Analysis

#### Frequencies of Botanicals and Applications

Frequencies and percentage of the total for of each variable were calculated to identify the most prevalent botanicals in each media format. These were then cross-tabulated against the applications of botanical products to examine and identify the most frequently mentioned applications for each type of botanical.

#### Comparison to Published Literature

Having identified the most common health applications associated with each of the most frequently mentioned botanicals, a PubMed search was conducted seeking published reviews supporting or refuting these claims. The search terms were the names of each of the botanical products or product groups individually and paired with their applications. Where no systematic reviews were available, single research studies were sought under the same search terms.

## Results

### Frequency of Herbs, Spices, and Other Botanicals

A total of 237 botanical products were identified. Many appeared only once or twice, and others appeared much more frequently. There were 128 mentions of botanical products in the print issues examined, with nearly 10 times as many (1215) on the websites. Appendix 1 includes an alphabetized list of all identified botanical products, with totals and percentages for both print and website materials. On the basis of this, the overall popularity of these products was assessed.

Some differences were noted between the frequency of mentions in print issues and web issues. The most frequently mentioned items in the print issues were black tea, green tea, ginger, cannabidiol, hemp seed, and oats. The most frequently mentioned items in the website issues were aloe vera, argan, ashwagandha, cannabidiol, chamomile, ginger, green tea, jojoba, lavender, oats, peppermint, rosemary, tea tree, and turmeric. A total of 2.5% (6/237) of the items emerged as the most frequently mentioned overall. These were aloe vera, ashwagandha, ginger, green tea, lavender, and turmeric.

### Health Applications and Methods of Use

In total, 18 unique health applications were identified as described previously and used to categorize the data. To identify the emphasis of health applications to each readership group, these were tabulated across source periodicals. Data for print editions are shown in Table 1, and data for website editions are shown in Table 2. Within the smaller number of print issues, skin care in *O, The Oprah Magazine* and energy increase in *Men’s Journal* had the highest frequency of mentions, consistent with the overall interests of their respective readerships.

The websites showed both similarities and differences to the printed editions. The websites for *Men’s Journal* and *Vogue* had significantly larger numbers of mentions of botanical products for health applications. In contrast to the print issues, immunity enhancement was the second most frequently mentioned health application overall after skin care, with stress reduction being the third most frequent. The most common applications mentioned on the *Men’s Journal* website were energy increase, stress reduction, and weight loss, and the most common applications mentioned on the *Vogue* website were immunity enhancement, stress reduction, and skin care. Immunity enhancement and stress reduction were also mentioned frequently in *Essence*; *Men’s Journal*; and *O, The Oprah Magazine*. This increasing level of interest in these applications was likely related to the increased concerns of readers during the emerging COVID-19 pandemic. Because of the faster production times for online content, these applications were mentioned first on the websites and later on in print issues.

To see how the various botanical products were being used, all health applications were examined by method of use, including ingested, topical, or airborne. These were compared for print and website editions as shown in Table 3.

**Table 1:** Frequencies of 18 identified health applications appearing in print issues of 10 periodicals studied from January 2020 to December 2020 (N=74).

	*The Advocate* (n=0), n (%)	*Elle* (n=9), n (%)	*Essence* (n=4), n (%)	*GQ* (n=12), n (%)	*Men’s Journal* (n=18), n (%)	*O, The Oprah Magazine* (n=15), n (%)	*Out* (n=2), n (%)	*Scientific American* (n=2), n (%)	*Time* (n=5), n (%)	*Vogue* (n=7), n (%)	Total, n (%)
Antimicrobial	0 (0)	0 (0)	0 (0)	0 (0)	0 (0)	0 (0)	0 (0)	0 (0)	0 (0)	0 (0)	0 (0)
Blood sugar management	0 (0)	0 (0)	0 (0)	0 (0)	0 (0)	0 (0)	0 (0)	0 (0)	0 (0)	0 (0)	0 (0)
Cognitive	0 (0)	0 (0)	0 (0)	0 (0)	0 (0)	0 (0)	0 (0)	1 (50)	0 (0)	1 (14)	2 (3)
Dental	0 (0)	0 (0)	0 (0)	0 (0)	0 (0)	0 (0)	0 (0)	0 (0)	0 (0)	0 (0)	0
Detoxifying	0 (0)	0 (0)	0 (0)	1 (8)	0 (0)	0 (0)	1 (50)	0 (0)	0 (0)	0 (0)	2 (3)
Immunity	0 (0)	0 (0)	1 (25)	1 (8)	3 (17)	1 (7)	0 (0)	0 (0)	0 (0)	1 (14)	7 (9)
Probiotics	0 (0)	0 (0)	0 (0)	1 (8)	1 (6)	1 (7)	0 (0)	1 (50)	0 (0)	0 (0)	4 (5)
Hair	0 (0)	3 (33)	2 (50)	0 (0)	0 (0)	1 (7)	0 (0)	0 (0)	0 (0)	2 (29)	8 (11)
Hormone regulation	0 (0)	0 (0)	0 (0)	0 (0)	0 (0)	0 (0)	0 (0)	0 (0)	0 (0)	0 (0)	0 (0)
Energy increase	0 (0)	0 (0)	0 (0)	3 (25)	7 (39)	1 (7)	0 (0)	0 (0)	0 (0)	0 (0)	11 (15)
Pain relief	0 (0)	1 (11)	0 (0)	2 (17)	3 (17)	0 (0)	0 (0)	0 (0)	0 (0)	0 (0)	6 (8)
Cancer prevention	0 (0)	0 (0)	0 (0)	0 (0)	0 (0)	1 (7)	0 (0)	0 (0)	0 (0)	0 (0)	1 (1)
CVD^a^ prevention	0 (0)	0 (0)	0 (0)	0 (0)	0 (0)	2 (13)	0 (0)	0 (0)	4 (80)	0 (0)	6 (8)
Stress reduction	0 (0)	3 (33)	0 (0)	2 (17)	0 (0)	2 (13)	0 (0)	0 (0)	0 (0)	1 (14)	8 (11)
Sexual enhancement	0 (0)	0 (0)	0 (0)	0 (0)	0 (0)	0 (0)	0 (0)	0 (0)	0 (0)	0 (0)	0 (0)
Skin care	0 (0)	2 (22)	1 (25)	0 (0)	2 (11)	5 (33)	1 (50)	0 (0)	0 (0)	2 (29)	13 (18)
Sleep aid	0 (0)	0 (0)	0 (0)	0 (0)	0 (0)	0 (0)	0 (0)	0 (0)	0 (0)	0 (0)	0 (0)
Weight loss	0 (0)	0 (0)	0 (0)	2 (17)	2 (11)	1 (7)	0 (0)	0 (0)	1 (20)	0 (0)	6 (8)

^a^CVD: cardiovascular disease.

**Table 2:** Frequencies of 18 identified health applications appearing in website issues of 10 periodicals studied from January 2020 to December 2020 (N=559).

	*The Advocate* (n=4), n (%)	*Elle* (n=36), n (%)	*Essence* (n=55), n (%)	*GQ* (n=46), n (%)	*Men’s Journal* (n=118), n (%)	*O, The Oprah Magazine* (n=50), n (%)	*Out* (n=10), n (%)	*Scientific American* (n=7), n (%)	*Time* (n=5), n (%)	*Vogue* (n=225), n (%)	Total, n (%)
Antimicrobial	0 (0)	1 (2.8)	2 (3.6)	3 (6.5)	4 (3.4)	4 (8)	0 (0)	1 (14.3)	1 (20)	18 (8)	34 (6.1)
Blood sugar management	0 (0)	0 (0)	2 (3.6)	0 (0)	3 (2.5)	0 (0)	0 (0)	0 (0)	0 (0)	0 (0)	5 (0.9)
Cognitive	0 (0)	1 (2.8)	3 (5.5)	2 (4.3)	4 (3.4)	0 (0)	0 (0)	0 (0)	0 (0)	5 (2.2)	15 (2.7)
Dental	0 (0)	1 (2.8)	0 (0)	1 (2.2)	0 (0)	0 (0)	0 (0)	0 (0)	0 (0)	0 (0)	2 (0.4)
Detoxifying	0 (0)	1 (2.8)	3 (5.5)	3 (6.5)	3 (2.5)	0 (0)	0 (0)	0 (0)	0 (0)	8 (3.6)	18 (3.2)
Immunity	1 (25)	5 (13.9)	12 (21.8)	9 (19.6)	15 (12.7)	10 (20)	0 (0)	1 (14.3)	2 (40)	28 (12.4)	83 (14.8)
Probiotics	0 (0)	1 (2.8)	4 (7.3)	4 (8.7)	10 (8.5)	0 (0)	0 (0)	1 (14.3)	0 (0)	5 (2.2)	25 (4.5)
Hair	0 (0)	2 (5.6)	1 (1.8)	3 (6.5)	1 (0.8)	12 (24)	1 (10)	1 (14.3)	0 (0)	19 (8.4)	40 (7.2)
Hormone regulation	0 (0)	1 (2.8)	0 (0)	0 (0)	3 (2.5)	1 (2)	0 (0)	0 (0)	0 (0)	6 (2.7)	11 (2)
Energy increase	0 (0)	1 (2.8)	3 (5.5)	1 (2.2)	13 (11)	0 (0)	0 (0)	0 (0)	0 (0)	8 (3.6)	26 (4.7)
Pain relief	1 (25)	4 (11.1)	2 (3.6)	1 (2.2)	6 (5.1)	4 (8)	2 (20)	0 (0)	0 (0)	13 (5.8)	33 (5.9)
Cancer prevention	0 (0)	0 (0)	0 (0)	1 (2.2)	1 (0.8)	0 (0)	0 (0)	0 (0)	0 (0)	0 (0)	2 (0.4)
CVD^a^ prevention	0 (0)	1 (2.8)	2 (3.6)	0 (0)	8 (6.8)	0 (0)	0 (0)	0 (0)	0 (0)	6 (2.7)	17 (3)
Stress reduction	1 (25)	7 (19.4)	7 (12.7)	3 (6.5)	14 (11.9)	6 (12)	2 (20)	1 (14.3)	1 (20)	37 (16.4)	79 (14.1)
Sexual enhancement	0 (0)	0 (0)	0 (0)	0 (0)	0 (0)	0 (0)	0 (0)	0 (0)	0 (0)	2 (0.9)	2 (0.4)
Skin care	1 (25)	11 (30.6)	9 (16.4)	12 (26.1)	14 (11.9)	14 (28)	5 (50)	1 (14.3)	1 (20)	58 (25.8)	126 (22.5)
Sleep aid	0 (0)	2 (5.6)	2 (3.6)	2 (4.3)	4 (3.4)	0 (0)	0 (0)	1 (14.3)	1 (20)	12 (5.3)	24 (4.3)
Weight loss	0 (0)	0 (0)	1 (1.8)	1 (2.2)	15 (12.7)	0 (0)	0 (0)	0 (0)	0 (0)	0 (0)	17 (3)

^a^CVD: cardiovascular disease.

**Table 3:** Comparison of mentions of 18 identified health applications between print and website issues of 10 periodicals studied from January 2020 to December 2020 by each of 3 identified methods of use.

	Print issues, n (%)				Websites, n (%)		
	Ingested (n=53)	Topical (n=25)	Airborne (n=0)	Ingested (n=276)		Topical (n=343)	Airborne (n=157)
Antimicrobial	0 (0)	0 (0)	0 (0)	10 (3.6)		27 (7.9)	12 (7.6)
Blood sugar management	0 (0)	0 (0)	0 (0)	6 (2.2)		0 (0)	0 (0)
Cognitive	2 (3.8)	0 (0)	0 (0)	14 (5.1)		5 (1.5)	4 (2.5)
Dental	0 (0)	0 (0)	0 (0)	0 (0)		2 (0.6)	1 (0.6)
Detoxifying	1 (1.9)	1 (4)	0 (0)	10 (3.6)		8 (2.3)	3 (1.9)
Immunity	6 (11.3)	1 (4)	0 (0)	51 (18.5)		43 (12.5)	22 (14)
Probiotics	4 (7.5)	0 (0)	0 (0)	25 (9.1)		1 (0.3)	4 (2.5)
Hair	1 (1.9)	7 (28)	0 (0)	7 (2.5)		35 (10.2)	4 (2.5)
Hormone regulation	0 (0)	0 (0)	0 (0)	12 (4.3)		4 (1.2)	3 (1.9)
Energy increase	12 (22.6)	1 (4)	0 (0)	17 (6.2)		13 (3.8)	6 (3.8)
Pain relief	5 (9.4)	3 (12)	0 (0)	8 (2.9)		21 (6.1)	12 (7.6)
Cancer prevention	6 (11.3)	0 (0)	0 (0)	2 (0.7)		0 (0)	0 (0)
CVD^a^ prevention	1 (1.9)	0 (0)	0 (0)	14 (5.1)		4 (1.2)	4 (2.5)
Stress reduction	5 (9.4)	3 (12)	0 (0)	40 (14.5)		54 (15.7)	40 (25.5)
Sexual enhancement	0 (0)	0 (0)	0 (0)	3 (1.1)		0 (0)	0 (0)
Skin care	4 (7.5)	9 (36)	0 (0)	26 (9.4)		115 (33.5)	31 (19.7)
Sleep aid	0 (0)	0 (0)	0 (0)	15 (5.4)		10 (2.9)	10 (6.4)
Weight loss	6 (11.3)	0 (0)	0 (0)	16 (5.8)		1 (0.3)	1 (0.6)

^a^CVD: cardiovascular disease.

In articles in print editions, the most frequent methods of use were ingested foods and supplements for immunity enhancement and weight loss and topical creams, lotions, and oils to reduce stress. Items from advertising in print issues differed, with ingested products to energy increase and topically applied products for skin care being the most frequently mentioned.

Looking at the same data for the articles in website editions, the most frequently mentioned methods of use were topical products for skin care, followed by ingested products for immunity enhancement and stress reduction. These applications were repeated in the website advertisements, but the most frequently mentioned method of use overall was topical application, highlighting creams, lotions, and oils not only for skin care but also for immunity enhancements and stress reduction.

To identify which botanicals were associated with which uses, the list of herbs, spices, and other botanical products was grouped according to similar types of products based on common culinary or cosmetic applications and compared across all 18 health applications. A complete tabulation of these can be found in Multimedia Appendix 1. Product group totals were also compared for the same variables. The complete list of botanicals tabulated by applications is available in Multimedia Appendix 2.

Finally, because they included primarily topical applications of the products, the antimicrobial, detoxifying, hair growth and repair, and hormone regulation categories were merged into the category of *skin care*. The *blood sugar management* and *dental care* categories had the fewest items and were removed from further analysis. The overall results are shown in Table 4.

**Table 4:** Condensed botanical categories with the most prevalent individual items from total mentions in both print and website issues compared across 4 specific functions. Botanical categories and the most prevalent individual items were compared to the literature. Results of this are indicated as below.

Botanical category		Total articles and advertisements (n=1533), n (%)	Total mentions of functions^a^ (n=1975), n (%)	Skin care (hair, face, and body; n=913), n (%)	Cognitive function (stress and mental health; n=106), n (%)	Immunity enhancement (n=705), n (%)	GI^b^ health and probiotics (n=184), n (%)
Algae (all)		47 (3.1)	44 (2.2)	17 (1.9)	5 (4.7)	18 (2.6)	4 (2.2)
**Aromatics (all)**		126 (8.2)	171 (8.7)	88 (9.6)	11 (10.4)	62 (8.8)	10 (5.4)
	Tea tree	20 (1.3)	21 (1.1)	12 (1.3)^c^	1 (0.9)	6 (0.9)	2 (1.1)
**Berries (all)**		73 (4.8)	106 (5.4)	51 (5.6)	9 (8.5)^c^	38 (5.4)^c^	8 (4.3)
	Rose hip	21 (1.4)	34 (1.7)	19 (2.1)	2 (1.9)	12 (1.7)	1 (0.5)
**Culinary herbs (all)**		173 (11.3)	242 (12.3)	113 (12.4)	12 (11.3)	96 (13.6)	21 (11.4)
	Peppermint	22 (1.4)	38 (1.9)	19 (2.1)^d^	1 (0.9)	14 (2)^e^	4 (2.2)
	Rosemary	24 (1.6)	38 (1.9)	21 (2.3)^c^	1 (0.9)	15 (2.1)	1 (0.5)
**Culinary spices (all)**		165 (10.8)	223 (11.3)	83 (9.1)	14 (13.2)	86 (12.2)	40 (21.7)
	Ginger	41 (2.7)	59 (3)	17 (1.9)	5 (4.7)	24 (3.4)^c^	13 (7.1)^c^
	Turmeric	39 (2.5)	43 (2.2)	14 (1.5)	2 (1.9)	18 (2.6)^c^	9 (4.9)
**Floral (all)**		197 (12.9)	210 (10.6)	108 (11.8)	9 (8.5)	81 (11.5)	12 (6.5)
	Lavender	49 (3.2)	55 (2.8)	27 (3)^c^	4 (3.8)^c^	22 (3.1)^e^	2 (1.1)
	Rose	29 (1.9)	32 (1.6)	20 (2.2)	1 (0.9)	10 (1.4)	1 (0.5)
**Herbs: other (all)**		326 (21.3)	477 (24.2)	218 (23.9)	29 (27.4)	181 (25.7)	49 (26.6)
	Ashwagandha	25 (1.6)	41 (2.1)	16 (1.8)^e^	4 (3.8)^c^	18 (2.6)^f^	3 (1.6)
	Chamomile	32 (2.1)	33 (1.7)	16 (1.8)^c^	1 (0.9)	13 (1.8)^e^	3 (1.6)
Mushrooms (all)		36 (2.3)	62 (3.1)	20 (2.2)^e^	10 (9.4)	26 (3.7)^f^	6 (3.3)
**Oils or seeds (all)**		160 (10.4)	172 (8.7)	85 (9.3)	16 (15.1)	61 (8.7)	10 (5.4)
	Argan	23 (1.5)	34 (1.7)	26 (2.8)^c^	2 (1.9)	6 (0.9)	0 (0)
	CBD^g^	42 (2.7)	22 (1.1)	5 (0.5)	2 (1.9)^c^	15 (2.1)	0 (0)
	Hemp seed	23 (1.5)	27 (1.4)	13 (1.4)	4 (3.8)	10 (1.4)	0 (0)
	Jojoba	31 (2)	34 (1.7)	19 (2.1)^c^	2 (1.9)	13 (1.8)	0 (0)
Seaweeds or coastal plants (all)		35 (2.3)	45 (2.3)	23 (2.5)^c^	2 (1.9)	16 (2.3)	4 (2.2)
**Tea (all)**		58 (3.8)	59 (3)	24 (2.6)^c^	5 (4.7)	24 (3.4)^c^	6 (3.3)
	Black tea	9 (0.6)	3 (0.2)	1 (0.1)	1 (0.9)	1 (0.1)	0 (0)
	Green tea	39 (2.5)	40 (2)	16 (1.8)	4 (3.8)	16 (2.3)^c^	4 (2.2)
**Other products (all)**		137 (8.9)	164 (8.3)	83 (9.1)	5 (4.7)	62 (8.8)	14 (7.6)
	Aloe vera	47 (3.1)	61 (3.1)	34 (3.7)^c^	1 (0.9)	22 (3.1)^f^	4 (2.2)
	Oats	27 (1.8)	35 (1.8)	15 (1.6)^e^	2 (1.9)	13 (1.8)	5 (2.7)^c^

^a^Columns are not mutually exclusive.

^b^GI: gastrointestinal.

^c^Supported by the literature.

^d^Not supported.

^e^Some research support.

^f^Mixed results found.

^g^CBD: cannabidiol.

### Comparison to Published Literature

#### Botanicals for Skin Care

After consolidating the categories as mentioned previously, the most frequently mentioned application overall was skin care, with several individual products predominating in the mentions. Consistent with its long use, the reviews by Sadoyu et al [32] and Chelu et al [33] supported the role of aloe vera in skin care and wound healing.

Many herbs were noted to have verifiable applications for skin care. Chamomile was also supported as having a role in skin care by reviews from El Mihyaoui et al [34] and Dai et al [35].

A review by Mandlik Ingawale and Namdeo [36] described the pharmacological properties of ashwagandha, including antibacterial and antifungal activity, which have applications in skin care.

Batiha et al [37] reviewed studies of the bioactive components and pharmacological activity of lavender. Although the exact mechanisms are still not well understood, they concluded that topical lavender can be effective against bacteria, fungi, yeasts, and mold and has antioxidant properties, all of which make it a desirable ingredient in skin care products.

A review by de Macedo et al [38] found that there were many topical applications of rosemary in skin care and described the biochemical bases for its efficacy. Another review by Li Pomi et al [39] described the potential for dermatological uses of rosemary and pointed out the evidence for its efficacy as protection from UV sun damage and as an antibacterial agent. While rosemary is considered safe for topical uses and when ingested in small amounts, it may not be safe if taken orally in large amounts [40].

Although peppermint was frequently mentioned as a component of skin care products, evidence for an independent function in this capacity is limited. A review by Zhao et al [41] examined the role of peppermint essential oil in treating skin conditions but not for use with healthy skin in any capacity. The frequent mention of peppermint in products for skin care may be more esthetic than functional.

Tea as applied for skin care was examined in a review by Koch et al [42], reporting that it is beneficial for skin care, including antiaging, as well as hair care. Seaweed similarly had strong evidence supporting its traditional role in skin care, along with other health applications such as antiaging, anticancer, and anti-inflammatory preparations [43-46].

Plant oils were also reported as a significant component of skin care products. A review by Kairey et al [47] found evidence for multiple applications of tea tree oil in skin health, including acne, dermatitis, skin inflammation, and photodamage. They noted that side effects of topical tea tree oil were minimal in the trials reviewed.

The evidence for jojoba as a skin care product was very strong, as described in the reviews by Lin et al [48], Gad et al [49], and Blaak and Staib [50]. No significant side effects were noted by these authors.

Mechqoq et al [51] reviewed the literature on the chemical composition and uses of argan oil and reported on its extensive use as a skin moisturizer, as well as a treatment for skin irritation of various types. Their review noted antibacterial and antifungal actions without notable side effects. A study by Alsatari et al [52] found that topical application of argan oil was an effective treatment for diaper dermatitis in infants. A mouse study by Makbal et al [53] found anti-inflammatory properties in argan oil, with no toxic effects noted even at high doses.

Mushrooms were also included as an ingredient in many topical products. Paterska et al [54] conducted an extensive review of the literature on a range of mushroom varieties for potential antiaging properties. They noted that many in vitro studies have found mushrooms to contain bioactive compounds such as polysaccharides, terpenoids, polyphenols, and peptides that may be beneficial to skin health. However, these conclusions were limited by a lack of human trials.

A review by Mineroff and Jagdeo [55] looking specifically at *Tremella fuciformis*, or snow mushrooms, found in vitro and animal studies suggesting a role in protection from UV damage and wound healing. However, these were inconclusive due to a lack of human studies. While there seem to be some potential benefits and minimal identified side effects, skin care applications for mushrooms are not strongly supported by published literature.

Oats and oatmeal are common ingredients in skin care products, incorporated for a variety of purposes. Becker et al [56] conducted a review of the safety and efficacy information available from both research and industry sources. They concluded that, when used as per industry guidelines, oatmeal is safe for use in skin care products, with demonstrated efficacy for many, if not all, of its applications. Kim et al [57] studied the means by which oats are able to ameliorate skin irritation from contact dermatitis in mice. They found that a topical application reduced skin lesions through an immune-modulatory mechanism. Similar studies with human participants were not found.

#### Botanicals for Stress Management

Although not mentioned as frequently as some of the other functions, several botanical products were indicated for applications in cognitive well-being and stress management. A systematic review and meta-analysis by Akhgarjand et al [58] found overall efficacy of ashwagandha for both stress and anxiety. A recent clinical study by Leonard et al [59] found that ashwagandha supplementation improved measures of cognitive function and stress in healthy young people.

Lavender was mentioned frequently but not as commonly for stress management. This was despite evidence that it is beneficial for this function. A systematic review and meta-analysis by Donelli et al [60] also looked at lavender as a treatment for anxiety. They found 65 randomized controlled trials and 25 nonrandomized studies of lavender for treatment of anxiety. Their overall conclusions were that oral administration of lavender essential oil is a safe and effective treatment for anxiety. They were more equivocal about inhaled lavender because of the confounding factors in the included studies. A review by Yoo and Park [61] examined studies of the effects of lavender inhalation on anxiety across a variety of human studies in stressful settings. They identified 11 clinical trials, with 10 showing reduction of anxiety using lavender. Sayed et al [62], in another systematic review and meta-analysis, concluded that inhaled lavender is best for short-term interventions for acute anxiety but orally dosed lavender is better for longer-term treatment. None of these authors reported any significant side effects from either route of administration [62].

Cannabidiol, a cannabis derivative, was frequently mentioned in the legacy media in relation to stress management, although less frequently than for other purposes. It was examined for this purpose in a number of review articles [63-66]. While all of these authors found cannabidiol to be a promising therapy for stress and anxiety, they all also called for further research as to its efficacy in specific situations, as well as further understanding of its biochemical mechanisms.

Finally, while often touted for their nutritional value, berries, with their high content of polyphenols, are anti-inflammatory and have also been associated with decreased stress and improved quality of sleep, although the mechanisms of these potential benefits have not yet been determined [67-70].

#### Botanicals and Immunity

Multiple reviews have investigated the immune modulation properties of various botanical products. Included in those studies were many of the botanicals identified from mentions in our sample of legacy media. These included aloe vera, anise, ashwagandha, astragalus, bay leaf, bilberry, black pepper, cardamon, chamomile, cinnamon, clove, cumin, echinacea, frankincense, garlic, ginger, ginseng, green tea, lemon, licorice, mint, myrrh, oregano, sage, shiitake mushroom, tulsi, and turmeric [71-73].

There were also reviews about botanicals in the form of essential oils for uses benefiting immune function. Human studies of beneficial effects of essential oils on immune function were described in a review by Peterfalvi et al [74] These included bergamot, cypress, fennel, ginger, lavender, patchouli, pepper, rose, sandalwood, and sweet marjoram. In another review, antimicrobial effects of fennel, peppermint, pine, and thyme essential oils were reported [75]. However, Sindle and Martin [76] advised caution with direct applications of essential oils to the skin in view of incidence of atopic dermatitis in sensitive individuals.

In addition to these multiproduct reviews, there were also many discussions of specific botanicals for immunity support. aloe vera was found to have a positive effect on stress-induced immunosuppression in mice, but no comparable studies in humans were found in PubMed [77]. Guo and Mei [78] cautioned about internal use because of potential toxicity of some compounds contained in aloe. A review by Hęś et al [79] supported the use of aloe as an antioxidant, but these authors cautioned against ingestion of high doses of aloe due to its laxative effects.

A review looking at rosemary by Ahmed and Babakir-Mina [80] found that, while animal studies have shown potential immunological benefits, human studies on this are lacking. Regarding ashwagandha, a review by Mandlik Ingawale and Namdeo [36] described its pharmacological properties, which included immunological activity. They also noted that the studies they reviewed showed little or no toxicity when taken in typical doses. A randomized controlled study by Verma et al [81] found no evidence of toxicity in healthy young adults. However, some caution is warranted as a review by Philips et al [82] found harmful effects in patients with liver disease consistent with a toxic effect.

Ginger was also frequently mentioned in relation to products supporting immunity. The evidence for this is less equivocal than that for aloe, with multiple review articles supporting this application [83,84] and a biochemical theoretical model by Kadhim et al [85] showing potential interference of components of ginger with the spike protein of the COVID-19 virus.

Tea, both green and black, has been studied extensively in terms of its effects on immune function, as reported in multiple review articles, demonstrating efficacy against the common cold and influenza [86], autoimmune disorders [87], and potentially as prevention or adjunctive treatment of COVID-19 [88-90].

Mushrooms are a group of heterogeneous products, but collectively, they have a long history of multiple health applications. These properties were discussed in a review by Yadav and Negi [91] and included “anti-tumor, antioxidant, immunomodulating, radical scavenging, cholesterol-lowering, cardiovascular, antimicrobial, anti-inflammatory, hepatoprotective, detoxicating, anti-obesity, anti-diabetic, analgesic, and various other properties.” The mixed nature of various types of mushrooms makes it difficult to draw definitive overall conclusions, as seen with the mixed results of the studies reported in the reviews by Motta et al [92] and Uffelman et al [93]. Hong et al [94] reviewed literature looking specifically at mushrooms and cardiometabolic diseases. They found some improvements reported for cardiometabolic factors such as glucose control and lipid profiles but not for overall mortality from cardiovascular disease.

Finally, a review by Govers et al [95] found that berries as a class can play a positive role in supporting immunity.

#### Botanicals for Gastrointestinal Health

Oats and ginger were 2 products mentioned for gastrointestinal health for which supporting literature was found. Oats are known to be a good source of soluble fiber and β-glucans. The soluble fiber increases gastrointestinal transit time and slows absorption. A range of gastrointestinal disorders have been shown to improve with increased intake of β-glucans [96].

A review by Fabiano et al [97] explored the prebiotic effects of oat consumption on the composition of the gut microbiome. They noted that oats are a source of short-chain fatty acids, which have been shown to decrease inflammation. However, these properties must be interpreted with caution due to a lack of large-scale studies in humans.

Ginger has long been used as a folk remedy for gastrointestinal symptoms, with substantial evidence of efficacy [84]. A review and meta-analysis by Li et al [98] concluded that the overall preponderance of published literature indicates that ginger is a safe and effective treatment for managing nausea and vomiting from a variety of causes, including pregnancy and cancer chemotherapy.

## Discussion

### Principal Findings

Each step of this study yielded specific results that were then applied in subsequent steps. Initially, we found that there were differences in the type of content readers are exposed to when they choose to read magazines in print or on the web. These different exposures may ultimately affect their product choices. The number and types of botanicals mentioned as well as their applications were different between these media types and across the different publications. Health-related purposes of use were also varied across readership groups. Seeing this level of heterogeneity, it then became important to assess the accuracy of the information provided.

The list of botanicals was cross-tabulated with the health applications identified. Specific uses of the most prevalently mentioned botanical products were then compared to published scientific literature.

### Accuracy of Claims in Legacy Media

Looking at these most frequently mentioned products, we found that there was strong evidence to support the use of aloe vera in topical skin care applications, but its use for immune enhancement was more equivocal, with mixed results being found. Although cannabidiol was not mentioned frequently for cognitive function or stress, there was better evidence for this application than for the more prevalent application of immunity enhancement. Ginger was strongly supported in its role in immunity enhancement, along with evidence for its role in gastrointestinal health. Tea, specifically green tea, has been extensively studied for a variety of applications, and our results found evidence suggesting a role in supporting immunity. Lavender, primarily as an essential oil, was supported in its application for immunity enhancement and skin care and for its more common application in stress management. Turmeric was similar to ginger in both its popularity and evidence of a role in immunity enhancement.

The legacy media examined in this study provided both articles and advertisements in which a range of botanical products were mentioned in association with a variety of health applications. While analysis of every reference to health applications of botanicals was beyond the scope of this study, comparison of the most prevalent of these health associations to published research literature indicates that, overall, these health associations are consistent with current scientific reports about the health applications of botanical products. While some products had a greater degree of research support than others, truly egregious falsehoods were absent. Therefore, legacy media may be considered a credible source of information to readers about these topics.

While it may seem surprising that more misinformation was not found, especially in the online materials, fact checking and editorial oversight may help limit this in legacy media. In the face of extensive health misinformation from many other venues in social media, it seems that legacy media does, to some extent, justify the trust placed in it by its readership with content that is substantially in agreement with current scientific literature.

### Limitations

The generalizability of these findings is limited due to the sample size and the time-limited data collection period. Analyses including similar data from periodicals in languages other than English and over a longer period would facilitate further understanding of this area.

In-depth examination of the literature on all botanicals mentioned across all the periodicals studied was not possible. A review of research including less prevalent products may have found examples of inaccurate communications in legacy media to a greater extent than was found in this study.

### Comparison With Prior Work

#### Accuracy of Claims on Social Media

Many studies have examined the accuracy of health information available on the internet and social media platforms. Looking specifically at websites for herbal supplements, Owens et al [99] found that <3% of the websites they surveyed included references to scientific literature to support their claims. They also noted the presence of testimonials from product users on these sites without oversight by website administrators. Some of these testimonials make potentially misleading claims about these products as treatments or cures of diseases, again without citing any peer-reviewed evidence.

A series of articles by Ng et al [100-103] used the DISCERN instrument to look at online consumer information on dietary and herbal supplements as recommended for fatigue [100], weight loss [101], hypertension [102], and pain [103]. The overall results of these studies found a high degree of variability in the quality of information available on these websites, with commercial websites generally providing poorer-quality information than the websites professional organizations or health care institutions. In a similar study of dietary and herbal supplements for COVID-19, Ng et al [104] examined information provided on Twitter and found comparable levels of misinformation circulating even more quickly than via web pages. An earlier study from Swetland et al [105] had reported that >25% of tweets about herbs and dietary supplements contained inaccurate information. Those authors also noted that they found no association between the accuracy of the content of these tweets and the number of likes or retweets they received. This indicates that accuracy made no difference in the response to the information presented and shared. Australian Instagram posts about dietary supplements were similarly inaccurate [106]. This is of concern because information found on the internet was found to be the most common reason for starting the use of supplements, usually without consulting a physician or pharmacist [107].

Investigations of social media in languages other than English have also found misinformation. Kharbat and Abu Daabes [108] found that the quality of information about herbs and cancer treatment in Arabic on YouTube was inadequate to properly advise patients. A total of 3 studies looking at health information on TikTok videos in Chinese all found that the science-related content of these videos was of variable quality, with those contributed by for-profit organizations having the lowest information quality [109-111].

Inclusion of misinformation seems to be no bar to the popularity of the health information presented online. In total, 2 studies of health videos posted on YouTube, one about kidney disease [93] and one about diabetes [112], found that, based on the number of views and likes, misleading videos were actually more popular than those containing evidence-based health information.

#### Approaches to Addressing Inaccuracies

To address inaccuracies in health communications throughout the media landscape, it is important to have clear definitions of what is meant. El Mikati et al [113] described the distinctions between misinformation, disinformation, and malinformation. Each of these has its own root causes and motivation by creators, and thus, different approaches to management are needed.

It has been noted by many researchers in this area that inaccurate information of every type proliferates in areas where there is a dearth of information provided by health professionals on platforms used by the public. There have been calls for an increase in engaging educational content to be created for this purpose [99,108,110,114-116]. Joseph et al [117] refer to a “tug-of-war” between dissemination of evidence-based health information and the spread of misinformation online.

Infodemiology and infoveillance provide approaches to understanding online misinformation and to organizing and winning this proverbial tug of war. Infodemiology examines both the existing information on the web (the “supply side”) and the information needs of the web-searching public (the “demand side”) [118]. Infoveillance is the monitoring of these sources of information with the goal of obtaining insights about users’ prioritization and responses to health-related issues [15]. Having a clearer understanding of the full scope of information both as provided in the media and as sought by the public can help establish the means to address the issue of inaccurate health information.

Suarez-Lledo and Alvarez-Galvez [16] identified 4 knowledge gaps to be filled that align with public health infoveillance goals. These can be summarized as (1) identifying dominant trends, (2) understanding mechanisms of misinformation spread, (3) determining the impact of misinformation on health behaviors, and (4) developing communication strategies to reduce and mitigate the negative impacts of misinformation.

The World Health Organization also addressed these issues in their first infodemiology conference [119]. They called for the creation of systems for social listening, signal detection, and analysis to identify emerging information trends. To achieve this, they recommended building collaborative partnerships beyond the usual health system networks to include people in journalism, digital media developers, and community organizations to develop a multifaceted toolkit of strategies. They noted that the health information ecosystem includes both online and offline environments and the inclusion of both is necessary to reach all communities.

Strategies have been proposed to add some options to the needed toolkit. Ishizumi et al [120] discussed “upstream” activities such as improving health literacy in populations, prebunking through education, and enhanced presence on social media platforms. They also promote social listening as a means to identify information needs and build trust.

### Conclusions

Our findings are significant to public health in that they stand in contrast to the much higher rates of misinformation appearing on social media platforms. Furthermore, while previous discussions of infodemiology note that the information ecosystem includes both online and offline media, research across both domains can be challenging [118,119]. By conducting an analysis of legacy media including both print and web versions, this study can serve as an example of how this can be done for further research in other content areas. Research inclusive of a wider range of media domains may facilitate better responses to misinformation wherever it appears and guide educational efforts.

## Appendix

Multimedia Appendix 1 Frequencies and percentages of the total number of botanicals appearing in 10 examples of legacy media from January 2020 to December 2020.

Multimedia Appendix 2 All herbs, spices, and other botanicals by health application.

## Data Availability

The datasets generated during and analyzed during this study are available from the corresponding author on reasonable request.
