# Supplementary material for: Health-Related Messages About Herbs, Spices, and Other Botanicals Appearing in Print Issues and Websites of Legacy Media: Content Analysis and Evaluation
Source: JMIR Form Res. 2024 Dec 4;8:e63281. doi: 10.2196/63281 (PMC11656503; doi:10.2196/63281)
Supplement: Multimedia Appendix 1 [file formative_v8i1e63281_app1.docx]

**Appendix 1.** Frequencies and percentages of the total number of botanicals appearing in 10 examples of legacy media from January 2020 to December 2020.

| Item | Print (n=128), n (%) | Web (n=1215), n (%) |
| --- | --- | --- |
| Acacia | 0 (0) | 3 (0.2) |
| Acai | 0 (0) | 10 (0.8) |
| Acerola cherry | 0 (0) | 6 (0.5) |
| Agave | 0 (0) | 7 (0.6) |
| Alfalfa | 0 (0) | 4 (0.3) |
| Algae | 4 (3.1) | 17 (1.4) |
| Aloe | 4 (3.1) | 43 (3.5) |
| Amla oramalaki | 0 (0) | 6 (0.5) |
| Angelica | 0 (0) | 2 (0.2) |
| Anise | 1 (0.8) | 2 (0.2) |
| Argan | 1 (0.8) | 22 (1.8) |
| Arnica | 0 (0) | 6 (0.5) |
| Ashitaba | 0 (0) | 1 (0.1) |
| Ashwagandha | 2 (1.6) | 23 (1.9) |
| Astragalus | 0 (0) | 10 (0.8) |
| Bacopa | 0 (0) | 4 (0.3) |
| Bakuchiol | 0 (0) | 7 (0.6) |
| Balsam | 0 (0) | 1 (0.1) |
| Bamboo | 3 (2.3) | 7 (0.6) |
| Baobab | 0 (0) | 7 (0.6) |
| Barberry | 0 (0) | 2 (0.2) |
| Basil | 0 (0) | 2 (0.2) |
| Bay leaf | 1 (0.8) | 1 (0.1) |
| Bearberry (*uva-ursi*) | 0 (0) | 5 (0.4) |
| Bergamot | 0 (0) | 10 (0.8) |
| Bhringraj | 0 (0) | 1 (0.1) |
| Bilberry | 0 (0) | 5 (0.4) |
| Birch | 0 (0) | 2 (0.2) |
| Blackberry leaf | 0 (0) | 1 (0.1) |
| Black pepper | 2 (1.6) | 9 (0.7) |
| Black tea | 6 (4.7) | 3 (0.2) |
| Bladder wrack | 1 (0.8) | 4 (0.3) |
| *Boswellia* | 0 (0) | 2 (0.3) |
| Burdock | 0 (0) | 6 (0.5) |
| Butterbur | 0 (0) | 2 (0.2) |
| Cacao | 0 (0) | 9 (0.7) |
| Cactus flower | 0 (0) | 2 (0.2) |
| Calendula | 0 (0) | 14 (1.2) |
| California poppy | 0 (0) | 2 (0.2) |
| Camellia | 0 (0) | 2 (0.2) |
| Camphor | 0 (0) | 4 (0.3) |
| Camu-camu | 0 (0) | 4 (0.3) |
| Cape lilac | 0 (0) | 1 (0.1) |
| Cardamom | 3 (2.3) | 7 (0.6) |
| Cascara | 0 (0) | 1 (0.1) |
| Cassia | 0 (0) | 1 (0.1) |
| Castor seed oil | 2 (1.6) | 6 (0.5) |
| Catnip | 0 (0) | 1 (0.1) |
| Cat’s claw | 0 (0) | 1 (0.1) |
| Cayenne | 0 (0) | 5 (0.4) |
| CBD^a^ | 12 (9.4) | 30 (2.5) |
| Cedarwood oil | 0 (0) | 10 (0.8) |
| Celery seed | 0 (0) | 1 (0.1) |
| Chaga mushrooms | 0 (0) | 5 (0.4) |
| Chamomile | 2 (1.6) | 30 (2.5) |
| Cherry blossom | 0 (0) | 1 (0.1) |
| Chia | 1 (0.8) | 13 (1.1) |
| Chickweed | 0 (0) | 1 (0.1) |
| Chicory | 0 (0) | 2 (0.2) |
| Chili pepper | 0 (0) | 4 (0.3) |
| Chlorella | 4 (3.1) | 5 (0.4) |
| Chlorophyll | 0 (0) | 2 (0.2) |
| Chrysanthemum | 0 (0) | 1 (0.1) |
| Cilantro | 0 (0) | 2 (0.2) |
| Cinnamon | 2 (1.6) | 9 (0.7) |
| Clary sage | 0 (0) | 4 (0.3) |
| Clove | 1 (0.8) | 4 (0.3) |
| Coffee | 1 (0.8) | 14 (1.2) |
| Comfrey | 0 (0) | 6 (0.5) |
| *Cordyceps* | 1 (0.8) | 7 (0.6) |
| Coriander | 0 (0) | 2 (0.2) |
| Cornflower | 0 (0) | 2 (0.2) |
| Cress | 0 (0) | 4 (0.3) |
| Crocus | 0 (0) | 1 (0.1) |
| Cumin | 1 (0.8) | 2 (0.2) |
| Cypress | 0 (0) | 5 (0.4) |
| Damiana leaf | 0 (0) | 2 (0.2) |
| Dandelion | 0 (0) | 8 (0.7) |
| Dang gui | 0 (0) | 1 (0.1) |
| Duckweed | 0 (0) | 1 (0.1) |
| Echinacea | 1 (0.8) | 6 (0.5) |
| Edelweiss | 0 (0) | 6 (0.5) |
| Elderflower | 1 (0.8) | 3 (0.2) |
| Eleuthero | 0 (0) | 1 (0.1) |
| Eucalyptus | 1 (0.8) | 17 (1.4) |
| *Eucommia* | 0 (0) | 1 (0.1) |
| Evening primrose | 0 (0) | 4 (0.3) |
| Fennel | 0 (0) | 4 (0.3) |
| Fenugreek | 0 (0) | 1 (0.1) |
| Feverfew | 0 (0) | 4 (0.3) |
| Flaxseed | 1 (0.8) | 10 (0.8) |
| Four o’clock flower | 0 (0) | 1 (0.1) |
| Frankincense | 1 (0.8) | 11 (0.9) |
| *Garcinia cambogia* | 0 (0) | 3 (0.2) |
| Gardenia | 0 (0) | 5 (0.4) |
| Garlic | 2 (1.6) | 3 (0.2) |
| Geranium | 0 (0) | 10 (0.8) |
| Ginger | 11 (8.6) | 30 (2.5) |
| Gingko | 0 (0) | 5 (0.4) |
| Ginseng | 0 (0) | 11 (0.9) |
| Goji berry | 0 (0) | 13 (1.1) |
| Goldthread | 0 (0) | 1 (0.1) |
| Gotu kola | 0 (0) | 4 (0.3) |
| Green tea | 5 (3.9) | 34 (2.8) |
| Guaraná | 0 (0) | 3 (0.2) |
| Heal-all | 0 (0) | 1 (0.1) |
| Hemp seed | 5 (3.9) | 18 (1.5) |
| Hibiscus | 0 (0) | 8 (0.7) |
| Holy basil | 0 (0) | 5 (0.41) |
| Honeysuckle | 0 (0) | 1 (0.08) |
| Hops | 1 (0.8) | 9 (0.7) |
| Horny goat weed | 0 (0) | 1 (0.1) |
| Horsetail | 0 (0) | 7 (0.6) |
| Hydrangea leaf | 0 (0) | 1 (0.1) |
| Indigo | 0 (0) | 1 (0.1) |
| Irish moss | 0 (0) | 2 (0.2) |
| *Isatis* (woad) | 0 (0) | 3 (0.2) |
| Japanese knotweed | 0 (0) | 3 (0.2) |
| Jasmine | 2 (1.6) | 7 (0.6) |
| Jojoba | 1 (0.8) | 30 (2.5) |
| Juniper | 0 (0) | 4 (0.3) |
| Laurel | 0 (0) | 2 (0.2) |
| Lavender | 1 (0.8) | 48 (4) |
| Lemon | 1 (0.8) | 16 (1.3) |
| Lemon balm | 0 (0) | 18 (1.5) |
| Lemongrass | 1 (0.8) | 4 (0.3) |
| Licorice root | 1 (0.8) | 16 (1.3) |
| Lime flower | 0 (0) | 1 (0.1) |
| Lion’s mane | 3 (2.3) | 6 (0.5) |
| Longan | 0 (0) | 1 (0.1) |
| Lotus | 0 (0) | 4 (0.3) |
| Lupine | 0 (0) | 1 (0.1) |
| Maca | 2 (1.6) | 3 (0.2) |
| Magnolia | 0 (0) | 3 (0.2) |
| Maidenhair | 0 (0) | 2 (0.2) |
| Maitake mushroom | 0 (0) | 2 (0.2) |
| Manuka | 0 (0) | 2 (0.2) |
| Maqui berry | 0 (0) | 1 (0.1) |
| Marigold | 1 (0.8) | 4 (0.3) |
| Marshmallow root | 0 (0) | 10 (0.8) |
| Matcha green tea | 0 (0) | 8 (0.7) |
| Meadowfoam | 0 (0) | 12 (1) |
| Mexican poppy | 0 (0) | 2 (0.2) |
| Milk thistle | 0 (0) | 5 (0.4) |
| Mimosa flower | 0 (0) | 1 (0.1) |
| Moringa | 0 (0) | 7 (0.6) |
| Motherwort | 0 (0) | 1 (0.1) |
| Mullein | 0 (0) | 1 (0.1) |
| Mustard | 0 (0) | 1 (0.1) |
| Myrtle | 0 (0) | 4 (0.3) |
| Nasturtium | 0 (0) | 1 (0.1) |
| Neem leaf | 0 (0) | 5 (0.4) |
| Neroli | 0 (0) | 8 (0.7) |
| Nettle | 0 (0) | 8 (0.7) |
| Noni | 0 (0) | 2 (0.2) |
| Nutmeg | 0 (0) | 2 (0.2) |
| Oats | 6 (4.7) | 21 (1.7) |
| Oat straw | 0 (0) | 1 (0.1) |
| Orchid | 0 (0) | 1 (0.1) |
| Oregano | 1 (0.8) | 6 (0.5) |
| Palmarosa | 0 (0) | 1 (0.1) |
| Palm/palm oil | 0 (0) | 4 (0.3) |
| Palo santo | 0 (0) | 3 (0.2) |
| Pansy | 0 (0) | 1 (0.1) |
| Parsley | 0 (0) | 1 (0.1) |
| Passionflower | 0 (0) | 6 (0.5) |
| Patchouli | 0 (0) | 4 (0.3) |
| Peony | 0 (0) | 1 (0.1) |
| Peppermint | 0 (0) | 22 (1.8) |
| Pine | 0 (0) | 8 (0.7) |
| Plantain | 0 (0) | 3 (0.2) |
| Psyllium husk | 0 (0) | 2 (0.2) |
| Purslane | 0 (0) | 4 (0.3) |
| Quandong | 0 (0) | 1 (0.1) |
| Raspberry leaf | 0 (0) | 2 (0.2) |
| *Rauvolfia* | 0 (0) | 2 (0.2) |
| Ravintsara | 0 (0) | 2 (0.2) |
| Red clover | 0 (0) | 2 (0.2) |
| Red pepper | 0 (0) | 1 (0.1) |
| Reishi mushroom | 1 (0.8) | 9 (0.7) |
| *Rhodiola* | 0 (0) | 11 (0.9) |
| Rooibos | 1 (0.8) | 1 (0.1) |
| Rose | 3 (2.3) | 26 (2.1) |
| Rose hip | 0 (0) | 21 (1.7) |
| Rosemary | 0 (0) | 24 (2) |
| Rose of Jericho | 0 (0) | 1 (0.1) |
| Rosewood | 0 (0) | 2 (0.2) |
| Saffron | 0 (0) | 2 (0.2) |
| Sage | 0 (0) | 10 (0.8) |
| St John’s wort | 0 (0) | 2 (0.2) |
| Salvia | 0 (0) | 1 (0.1) |
| Sandalwood | 0 (0) | 9 (0.7) |
| Saw palmetto | 0 (0) | 3 (0.2) |
| *Schisandra* | 0 (0) | 5 (0.4) |
| Sea buckthorn | 0 (0) | 8 (0.7) |
| Sea fennel | 0 (0) | 3 (0.2) |
| Sea kelp | 2 (1.6) | 7 (0.6) |
| Sea moss | 0 (0) | 1 (0.1) |
| Seaweed | 2 (1.6) | 5 (0.4) |
| Senna | 0 (0) | 3 (0.2) |
| Shatavari | 0 (0) | 2 (0.2) |
| Shepherd’s purse | 0 (0) | 1 (0.1) |
| Shilajit | 0 (0) | 1 (0.1) |
| Shiso | 0 (0) | 1 (0.1) |
| Shiitake mushroom | 0 (0) | 1 (0.1) |
| Skullcap | 0 (0) | 5 (0.4) |
| Spearmint | 0 (0) | 5 (0.4) |
| Speedwell | 0 (0) | 2 (0.2) |
| Spirulina | 4 (3.1) | 7 (0.6) |
| Spruce | 0 (0) | 1 (0.1) |
| Stevia | 1 (0.8) | 1 (0.1) |
| Sugarcane | 0 (0) | 9 (0.7) |
| Sweetgrass | 0 (0) | 1 (0.1) |
| Tansy | 0 (0) | 2 (0.2) |
| Tarragon | 0 (0) | 2 (0.2) |
| Tea tree | 0 (0) | 20 (1.6) |
| Thistle | 0 (0) | 1 (0.1) |
| Thyme | 0 (0) | 7 (0.6) |
| *Tremella* mushroom | 0 (0) | 1 (0.1) |
| Tuberose | 0 (0) | 1 (0.1) |
| Tulsi | 0 (0) | 4 (0.3) |
| Turmeric | 4 (3.1) | 35 (2.9) |
| Valerian | 0 (0) | 7 (0.6) |
| Vanilla | 1 (0.8) | 4 (0.3) |
| Vetiver | 0 (0) | 5 (0.4) |
| Violet | 0 (0) | 2 (0.2) |
| Wakame | 1 (0.8) | 1 (0.1) |
| White tea | 0 (0) | 2 (0.2) |
| Willow bark | 1 (0.8) | 8 (0.7) |
| Witch hazel | 1 (0.8) | 10 (0.8) |
| Wormwood | 0 (0) | 1 (0.1) |
| Yarrow | 0 (0) | 3 (0.2) |
| Yaupon | 0 (0) | 1 (0.1) |
| Yerba santa | 0 (0) | 1 (0.1) |
| Yohimbe | 2 (1.6) | 1 (0.1) |
| Ylang-ylang | 0 (0) | 3 (0.2) |
| Yerba mate | 0 (0) | 2 (0.2) |

^a^CBD: cannabidiol.
