# Supplementary material for: Health-Related Messages About Herbs, Spices, and Other Botanicals Appearing in Print Issues and Websites of Legacy Media: Content Analysis and Evaluation
Source: JMIR Form Res. 2024 Dec 4;8:e63281. doi: 10.2196/63281 (PMC11656503; doi:10.2196/63281)
Supplement: Multimedia Appendix 2 [file formative_v8i1e63281_app2.docx]

Appendix 2. All herbs, spices and other botanicals by health applications

| Botanical | Total Articles and Ads | Antimicrobial | Blood sugar management | Cognitive function | Dental care | Detoxifying |
| --- | --- | --- | --- | --- | --- | --- |
| algae (all) | 41 | 5 | 1 | 5 | 1 | 9 |
| algae | 21 | 3 | 1 | 2 | 1 | 4 |
| chlorella | 9 | 1 | 0 | 1 | 0 | 2 |
| spirulina | 11 | 1 | 0 | 2 | 0 | 3 |
| aromatics (all) | 126 | 43 | 0 | 11 | 3 | 10 |
| acacia | 3 | 1 | 0 | 0 | 0 | 1 |
| balsam | 1 | 1 | 0 | 0 | 0 | 0 |
| camphor | 4 | 0 | 0 | 0 | 0 | 0 |
| cedarwood oil | 10 | 6 | 0 | 2 | 0 | 1 |
| cypress | 5 | 2 | 0 | 0 | 0 | 0 |
| eucalyptus | 18 | 6 | 0 | 3 | 1 | 1 |
| frankincense | 12 | 7 | 0 | 1 | 0 | 0 |
| juniper | 4 | 2 | 0 | 1 | 0 | 0 |
| lemon | 17 | 5 | 0 | 1 | 0 | 1 |
| myrrh | 8 | 1 | 0 | 0 | 0 | 1 |
| palmarosa | 1 | 0 | 0 | 0 | 0 | 0 |
| pine | 8 | 3 | 0 | 1 | 0 | 1 |
| sandalwood | 9 | 2 | 0 | 1 | 0 | 1 |
| spruce | 1 | 0 | 0 | 0 | 0 | 0 |
| tea tree | 20 | 4 | 0 | 1 | 2 | 1 |
| vetiver | 5 | 3 | 0 | 0 | 0 | 2 |
| berries (All) | 73 | 14 | 0 | 9 | 5 | 12 |
| acai | 10 | 0 | 0 | 2 | 0 | 1 |
| acerola cherry | 6 | 0 | 0 | 4 | 1 | 2 |
| amla / amalaki | 6 | 1 | 0 | 1 | 0 | 0 |
| barberry | 2 | 0 | 0 | 0 | 0 | 1 |
| bearberry (uva ursi) | 5 | 1 | 0 | 0 | 0 | 1 |
| bilberry | 5 | 1 | 0 | 0 | 0 | 0 |
| camu camu | 4 | 0 | 0 | 0 | 1 | 1 |
| goji berry | 13 | 3 | 0 | 0 | 1 | 2 |
| maqui berry | 1 | 0 | 0 | 0 | 0 | 0 |
| rose hip | 21 | 8 | 0 | 2 | 2 | 4 |
| culinary herbs (all) | 173 | 49 | 0 | 12 | 10 | 20 |
| angelica | 6 | 0 | 0 | 0 | 0 | 0 |
| basil | 2 | 1 | 0 | 0 | 0 | 0 |
| bay leaf / laurel | 4 | 0 | 0 | 1 | 0 | 0 |
| bergamot | 10 | 4 | 0 | 2 | 1 | 1 |
| chicory | 2 | 0 | 0 | 0 | 0 | 1 |
| cilantro | 2 | 2 | 0 | 1 | 0 | 1 |
| clary sage | 4 | 0 | 0 | 0 | 1 | 1 |
| cress | 4 | 1 | 0 | 0 | 0 | 0 |
| fennel | 4 | 0 | 0 | 0 | 0 | 1 |
| fenugreek | 1 | 0 | 0 | 0 | 0 | 0 |
| holy basil /  Tulsi | 9 | 0 | 0 | 0 | 0 | 3 |
| hops | 10 | 0 | 0 | 0 | 0 | 1 |
| lemon balm | 19 | 2 | 0 | 0 | 0 | 2 |
| lemongrass | 5 | 1 | 0 | 0 | 0 | 2 |
| lime flower | 1 | 0 | 0 | 0 | 0 | 0 |
| oregano | 7 | 3 | 0 | 2 | 0 | 1 |
| parsley | 1 | 0 | 0 | 0 | 0 | 0 |
| peppermint | 22 | 10 | 0 | 1 | 1 | 2 |
| rosemary | 24 | 13 | 0 | 1 | 3 | 1 |
| saffron | 2 | 1 | 0 | 0 | 0 | 0 |
| sage | 10 | 2 | 0 | 3 | 2 | 2 |
| spearmint | 5 | 2 | 0 | 0 | 1 | 0 |
| tarragon | 2 | 0 | 0 | 0 | 0 | 0 |
| stevia | 2 | 0 | 0 | 0 | 0 | 1 |
| sugarcane | 9 | 3 | 0 | 0 | 1 | 0 |
| thyme | 7 | 4 | 0 | 1 | 0 | 0 |
| culinary spices (all) | 165 | 21 | 2 | 14 | 1 | 23 |
| anise | 3 | 0 | 0 | 0 | 0 | 1 |
| black pepper | 11 | 1 | 0 | 1 | 0 | 1 |
| cacao | 9 | 0 | 0 | 0 | 1 | 3 |
| cardamom | 10 | 3 | 0 | 1 | 0 | 0 |
| celery seed | 1 | 0 | 0 | 0 | 0 | 0 |
| cinnamon | 11 | 3 | 0 | 1 | 0 | 2 |
| clove | 5 | 1 | 0 | 0 | 0 | 0 |
| coriander | 2 | 1 | 0 | 1 | 0 | 1 |
| cumin | 3 | 0 | 0 | 1 | 0 | 0 |
| garlic | 5 | 1 | 0 | 1 | 0 | 0 |
| ginger | 41 | 4 | 1 | 5 | 0 | 5 |
| licorice root | 17 | 1 | 0 | 0 | 0 | 4 |
| mustard | 1 | 0 | 0 | 0 | 0 | 0 |
| nutmeg | 2 | 1 | 0 | 0 | 0 | 0 |
| turmeric | 39 | 3 | 0 | 2 | 0 | 5 |
| vanilla | 5 | 1 | 0 | 0 | 0 | 0 |
| peppers (all) | 10 | 1 | 1 | 1 | 0 | 1 |
| cayenne | 5 | 1 | 1 | 1 | 0 | 1 |
| chili pepper | 4 | 0 | 0 | 0 | 0 | 0 |
| red pepper | 1 | 0 | 0 | 0 | 0 | 0 |
| floral (all) | 197 | 42 | 1 | 9 | 6 | 18 |
| cactus flower | 2 | 0 | 0 | 0 | 1 | 1 |
| calendula | 14 | 6 | 1 | 0 | 0 | 1 |
| California poppy | 2 | 0 | 0 | 0 | 0 | 0 |
| camellia | 2 | 0 | 0 | 0 | 0 | 0 |
| cape lilac | 1 | 0 | 0 | 0 | 0 | 0 |
| cherry blossom | 1 | 0 | 0 | 0 | 0 | 0 |
| chrysanthemum | 1 | 0 | 0 | 0 | 0 | 0 |
| cornflower | 2 | 0 | 0 | 0 | 0 | 0 |
| crocus | 1 | 0 | 0 | 0 | 0 | 0 |
| edelweiss | 6 | 0 | 0 | 0 | 0 | 0 |
| elderflower | 4 | 0 | 0 | 0 | 0 | 0 |
| four o’ clock | 1 | 0 | 0 | 0 | 0 | 0 |
| gardenia | 5 | 1 | 0 | 0 | 1 | 1 |
| geranium | 10 | 4 | 0 | 1 | 0 | 0 |
| hibiscus | 8 | 1 | 0 | 0 | 0 | 0 |
| honeysuckle | 1 | 0 | 0 | 0 | 0 | 1 |
| jasmine | 9 | 1 | 0 | 1 | 0 | 1 |
| lavender | 49 | 12 | 0 | 4 | 1 | 5 |
| lotus | 4 | 2 | 0 | 0 | 0 | 0 |
| lupine | 1 | 0 | 0 | 0 | 0 | 0 |
| magnolia | 3 | 0 | 0 | 0 | 0 | 1 |
| marigold | 5 | 1 | 0 | 0 | 0 | 0 |
| manuka | 2 | 0 | 0 | 0 | 0 | 1 |
| Mexican poppy | 2 | 0 | 0 | 0 | 0 | 0 |
| mimosa flower | 1 | 1 | 0 | 0 | 1 | 0 |
| nasturtium | 1 | 0 | 0 | 0 | 0 | 0 |
| neroli | 8 | 1 | 0 | 0 | 0 | 0 |
| orchid | 1 | 0 | 0 | 0 | 0 | 0 |
| pansy | 1 | 0 | 0 | 0 | 0 | 0 |
| passionflower | 6 | 0 | 0 | 0 | 0 | 1 |
| patchouli | 4 | 2 | 0 | 2 | 0 | 1 |
| peony | 1 | 0 | 0 | 0 | 0 | 0 |
| rose | 29 | 8 | 0 | 1 | 2 | 3 |
| salvia | 1 | 0 | 0 | 0 | 0 | 0 |
| tansy | 2 | 1 | 0 | 0 | 0 | 0 |
| tuberose | 1 | 0 | 0 | 0 | 0 | 0 |
| violet | 2 | 0 | 0 | 0 | 0 | 1 |
| ylang ylang | 3 | 1 | 0 | 0 | 0 | 0 |
| herbs – other (all) | 326 | 50 | 0 | 29 | 8 | 66 |
| arnica | 6 | 1 | 0 | 0 | 0 | 1 |
| ashitaba | 1 | 0 | 0 | 0 | 0 | 0 |
| ashwaganda | 25 | 4 | 0 | 4 | 1 | 4 |
| astragalus | 10 | 0 | 0 | 1 | 0 | 3 |
| bacopa | 4 | 0 | 0 | 3 | 0 | 0 |
| bakuchiol | 7 | 1 | 0 | 0 | 0 | 1 |
| bhringraj | 1 | 0 | 0 | 0 | 0 | 0 |
| blackberry leaf | 1 | 0 | 0 | 0 | 0 | 0 |
| boswellia | 2 | 0 | 0 | 1 | 0 | 0 |
| burdock | 6 | 1 | 0 | 1 | 0 | 4 |
| butterbur | 2 | 0 | 0 | 0 | 0 | 0 |
| cassia | 1 | 0 | 0 | 0 | 0 | 0 |
| catnip | 1 | 0 | 0 | 0 | 0 | 0 |
| cat's claw | 1 | 0 | 0 | 0 | 0 | 0 |
| chamomile | 32 | 6 | 0 | 1 | 1 | 5 |
| chickweed | 1 | 1 | 0 | 0 | 0 | 0 |
| comfrey | 6 | 3 | 0 | 0 | 0 | 1 |
| damiana leaf | 2 | 0 | 0 | 0 | 0 | 0 |
| dandelion | 8 | 0 | 0 | 1 | 0 | 2 |
| dang gui | 1 | 0 | 0 | 0 | 0 | 0 |
| duckweed | 1 | 0 | 0 | 0 | 0 | 1 |
| echinacea | 7 | 1 | 0 | 1 | 0 | 2 |
| eleuthero | 1 | 0 | 0 | 0 | 0 | 0 |
| eucommia | 1 | 0 | 0 | 0 | 0 | 0 |
| feverfew | 4 | 0 | 0 | 0 | 0 | 1 |
| garcinia cambogia | 3 | 1 | 0 | 0 | 0 | 2 |
| gingko | 5 | 0 | 0 | 3 | 0 | 0 |
| ginseng | 11 | 1 | 0 | 0 | 0 | 1 |
| goldthread | 1 | 0 | 0 | 0 | 0 | 0 |
| gotu kola | 4 | 0 | 0 | 0 | 0 | 1 |
| guarana | 3 | 0 | 0 | 0 | 0 | 0 |
| heal-all | 1 | 0 | 0 | 0 | 0 | 0 |
| horny goat weed | 1 | 0 | 0 | 0 | 0 | 1 |
| horsetail | 7 | 1 | 0 | 0 | 1 | 1 |
| hydrangea leaf | 1 | 0 | 0 | 0 | 0 | 0 |
| indigo | 1 | 0 | 0 | 0 | 0 | 0 |
| Irish moss | 2 | 0 | 0 | 0 | 0 | 0 |
| isatis (woad) | 3 | 1 | 0 | 0 | 0 | 2 |
| Japanese knotweed | 3 | 0 | 0 | 0 | 0 | 0 |
| longan | 1 | 0 | 0 | 0 | 0 | 1 |
| maca | 5 | 2 | 0 | 1 | 0 | 3 |
| maidenhair | 2 | 1 | 0 | 0 | 0 | 0 |
| marshmallow root | 10 | 3 | 0 | 1 | 2 | 1 |
| meadowfoam | 12 | 2 | 0 | 0 | 0 | 0 |
| milk thistle | 5 | 0 | 0 | 0 | 0 | 2 |
| moringa | 7 | 0 | 0 | 0 | 0 | 1 |
| motherwort | 1 | 0 | 0 | 0 | 0 | 0 |
| mullein | 1 | 0 | 0 | 1 | 0 | 1 |
| myrtle | 4 | 1 | 0 | 1 | 1 | 1 |
| neem leaf | 5 | 0 | 0 | 0 | 1 | 1 |
| nettle | 8 | 1 | 0 | 1 | 0 | 1 |
| noni | 2 | 0 | 0 | 0 | 0 | 2 |
| palo santo | 3 | 3 | 0 | 2 | 0 | 1 |
| plantain | 3 | 2 | 0 | 0 | 0 | 2 |
| quandong | 1 | 0 | 0 | 0 | 0 | 1 |
| raspberry leaf | 2 | 1 | 0 | 0 | 0 | 0 |
| rauwolfia | 2 | 0 | 0 | 0 | 0 | 0 |
| ravintsara | 2 | 0 | 0 | 0 | 0 | 0 |
| red clover | 2 | 0 | 0 | 0 | 0 | 2 |
| rhodiola | 11 | 0 | 0 | 2 | 0 | 2 |
| rooibos | 2 | 0 | 0 | 0 | 0 | 1 |
| rose of Jericho | 1 | 0 | 0 | 0 | 0 | 0 |
| saint John's wort | 2 | 1 | 0 | 0 | 0 | 0 |
| saw palmetto | 3 | 0 | 0 | 0 | 0 | 0 |
| schisandra | 5 | 0 | 0 | 0 | 0 | 3 |
| senna | 3 | 0 | 0 | 0 | 0 | 1 |
| shatavari | 2 | 0 | 0 | 1 | 0 | 1 |
| shepherd’s purse | 1 | 1 | 0 | 0 | 0 | 0 |
| skullcap | 5 | 0 | 0 | 0 | 0 | 0 |
| Solomon’s seal | 4 | 0 | 0 | 0 | 0 | 1 |
| speedwell | 2 | 0 | 0 | 0 | 0 | 0 |
| sweetgrass | 1 | 1 | 0 | 0 | 0 | 0 |
| thistle | 1 | 1 | 0 | 0 | 0 | 0 |
| valerian | 7 | 0 | 0 | 0 | 0 | 2 |
| witch hazel | 11 | 4 | 0 | 1 | 0 | 1 |
| wormwood | 1 | 1 | 0 | 0 | 0 | 0 |
| yarrow | 3 | 1 | 0 | 0 | 0 | 0 |
| yaupon | 1 | 1 | 0 | 1 | 0 | 0 |
| yerba santa | 1 | 0 | 0 | 1 | 0 | 1 |
| yohimbe | 3 | 0 | 0 | 0 | 0 | 0 |
| yerba mate | 2 | 1 | 0 | 0 | 1 | 0 |
| mushrooms (all) | 36 | 5 | 0 | 10 | 0 | 7 |
| chaga mushrooms | 5 | 0 | 0 | 0 | 0 | 0 |
| cordyceps | 8 | 3 | 0 | 2 | 0 | 2 |
| lion's mane | 9 | 0 | 0 | 5 | 0 | 1 |
| reishi mushroom | 10 | 1 | 0 | 3 | 0 | 4 |
| tremella mushroom | 1 | 0 | 0 | 0 | 0 | 0 |
| maitake mushroom | 2 | 1 | 0 | 0 | 0 | 0 |
| shitake mushroom | 1 | 0 | 0 | 0 | 0 | 0 |
| oils/seeds (all) | 160 | 18 | 0 | 16 | 4 | 7 |
| argan | 23 | 4 | 0 | 2 | 2 | 0 |
| castor seed oil | 8 | 0 | 0 | 0 | 0 | 0 |
| CBD | 42 | 3 | 0 | 2 | 0 | 1 |
| chia | 14 | 1 | 0 | 3 | 0 | 1 |
| evening primrose | 4 | 0 | 0 | 0 | 1 | 1 |
| flaxseed | 11 | 0 | 0 | 3 | 0 | 1 |
| hemp seed | 23 | 4 | 0 | 4 | 0 | 2 |
| jojoba | 31 | 6 | 0 | 2 | 1 | 1 |
| palm/palm oil | 4 | 0 | 0 | 0 | 0 | 0 |
| seaweeds (all) | 35 | 7 | 0 | 2 | 2 | 2 |
| bladderwrack | 5 | 1 | 0 | 0 | 0 | 0 |
| sea buckthorn | 8 | 3 | 0 | 1 | 0 | 1 |
| sea fennel | 3 | 1 | 0 | 0 | 1 | 0 |
| sea kelp | 9 | 1 | 0 | 0 | 1 | 0 |
| sea moss | 1 | 1 | 0 | 0 | 0 | 1 |
| seaweed | 7 | 0 | 0 | 1 | 0 | 0 |
| wakame | 2 | 0 | 0 | 0 | 0 | 0 |
| tea (all) | 58 | 2 | 2 | 5 | 3 | 8 |
| black tea | 9 | 0 | 0 | 1 | 0 | 0 |
| green tea | 39 | 1 | 2 | 4 | 2 | 6 |
| matcha green tea | 8 | 0 | 0 | 0 | 0 | 2 |
| white tea | 2 | 1 | 0 | 0 | 1 | 0 |
| other products (all) | 137 | 31 | 1 | 5 | 6 | 16 |
| agave | 7 | 0 | 0 | 0 | 0 | 0 |
| aloe | 47 | 12 | 0 | 1 | 1 | 5 |
| bamboo | 10 | 3 | 0 | 0 | 1 | 0 |
| baobab | 7 | 1 | 0 | 0 | 0 | 0 |
| birch | 2 | 1 | 0 | 0 | 1 | 1 |
| cascara | 1 | 0 | 0 | 0 | 0 | 1 |
| chlorophyll | 2 | 0 | 0 | 0 | 0 | 0 |
| coffee | 15 | 5 | 0 | 2 | 0 | 3 |
| oats | 27 | 6 | 1 | 2 | 2 | 3 |
| oat straw | 1 | 0 | 0 | 0 | 0 | 0 |
| psyllium husk | 2 | 0 | 0 | 0 | 0 | 1 |
| purslane | 4 | 0 | 0 | 0 | 0 | 0 |
| rosewood | 2 | 1 | 0 | 0 | 0 | 0 |
| shilajit | 1 | 0 | 0 | 0 | 0 | 1 |
| willow bark | 9 | 2 | 0 | 0 | 1 | 1 |
| Botanical | Total Articles and Ads | Enhanced Immunity | GI Health Probiotic | Hair Growth & Repair | Hormone Regulation |  |
| algae (all) | 41 | 18 | 4 | 3 | 0 |  |
| algae | 21 | 10 | 2 | 2 | 0 |  |
| chlorella | 9 | 3 | 1 | 0 | 0 |  |
| spirulina | 11 | 5 | 1 | 1 | 0 |  |
| aromatics (all) | 126 | 62 | 10 | 30 | 5 |  |
| acacia | 3 | 0 | 1 | 1 | 0 |  |
| balsam | 1 | 0 | 0 | 0 | 0 |  |
| camphor | 4 | 2 | 0 | 0 | 0 |  |
| cedarwood oil | 10 | 5 | 1 | 6 | 0 |  |
| cypress | 5 | 3 | 0 | 1 | 0 |  |
| eucalyptus | 18 | 11 | 1 | 3 | 0 |  |
| frankincense | 12 | 7 | 0 | 2 | 0 |  |
| juniper | 4 | 1 | 0 | 0 | 0 |  |
| lemon | 17 | 12 | 3 | 5 | 1 |  |
| myrrh | 8 | 1 | 0 | 0 | 0 |  |
| palmarosa | 1 | 1 | 0 | 0 | 0 |  |
| pine | 8 | 6 | 1 | 0 | 2 |  |
| sandalwood | 9 | 2 | 0 | 3 | 1 |  |
| spruce | 1 | 1 | 0 | 0 | 0 |  |
| tea tree | 20 | 6 | 2 | 7 | 0 |  |
| vetiver | 5 | 4 | 1 | 2 | 1 |  |
| berries (All) | 73 | 38 | 8 | 23 | 2 |  |
| acai | 10 | 7 | 3 | 2 | 0 |  |
| acerola cherry | 6 | 0 | 0 | 2 | 0 |  |
| amla / amalaki | 6 | 4 | 2 | 3 | 1 |  |
| barberry | 2 | 0 | 1 | 1 | 0 |  |
| bearberry (uva ursi) | 5 | 1 | 1 | 0 | 0 |  |
| bilberry | 5 | 2 | 0 | 0 | 0 |  |
| camu camu | 4 | 1 | 0 | 2 | 0 |  |
| goji berry | 13 | 10 | 0 | 5 | 1 |  |
| maqui berry | 1 | 1 | 0 | 1 | 0 |  |
| rose hip | 21 | 12 | 1 | 7 | 0 |  |
| culinary herbs (all) | 173 | 96 | 21 | 32 | 12 |  |
| angelica | 6 | 0 | 0 | 1 | 0 |  |
| basil | 2 | 1 | 0 | 1 | 0 |  |
| bay leaf / laurel | 4 | 4 | 1 | 0 | 1 |  |
| bergamot | 10 | 5 | 2 | 4 | 0 |  |
| chicory | 2 | 0 | 1 | 0 | 0 |  |
| cilantro | 2 | 2 | 0 | 0 | 0 |  |
| clary sage | 4 | 1 | 0 | 0 | 0 |  |
| cress | 4 | 3 | 0 | 1 | 1 |  |
| fennel | 4 | 2 | 3 | 0 | 0 |  |
| fenugreek | 1 | 0 | 0 | 1 | 0 |  |
| holy basil /  Tulsi | 9 | 6 | 1 | 1 | 2 |  |
| hops | 10 | 5 | 2 | 1 | 2 |  |
| lemon balm | 19 | 6 | 2 | 1 | 0 |  |
| lemongrass | 5 | 2 | 0 | 2 | 0 |  |
| lime flower | 1 | 1 | 0 | 0 | 0 |  |
| oregano | 7 | 6 | 1 | 0 | 1 |  |
| parsley | 1 | 1 | 0 | 0 | 0 |  |
| peppermint | 22 | 14 | 4 | 6 | 1 |  |
| rosemary | 24 | 15 | 1 | 7 | 0 |  |
| saffron | 2 | 2 | 0 | 1 | 0 |  |
| sage | 10 | 7 | 1 | 3 | 2 |  |
| spearmint | 5 | 3 | 0 | 1 | 0 |  |
| tarragon | 2 | 0 | 0 | 0 | 0 |  |
| stevia | 2 | 1 | 0 | 0 | 1 |  |
| sugarcane | 9 | 3 | 0 | 2 | 0 |  |
| thyme | 7 | 5 | 2 | 2 | 1 |  |
| culinary spices (all) | 165 | 86 | 40 | 23 | 16 |  |
| anise | 3 | 0 | 1 | 0 | 0 |  |
| black pepper | 11 | 4 | 1 | 4 | 1 |  |
| cacao | 9 | 6 | 1 | 1 | 1 |  |
| cardamom | 10 | 5 | 0 | 3 | 0 |  |
| celery seed | 1 | 1 | 0 | 0 | 0 |  |
| cinnamon | 11 | 6 | 5 | 1 | 1 |  |
| clove | 5 | 3 | 0 | 1 | 0 |  |
| coriander | 2 | 2 | 1 | 1 | 0 |  |
| cumin | 3 | 3 | 2 | 0 | 1 |  |
| garlic | 5 | 3 | 3 | 0 | 1 |  |
| ginger | 41 | 24 | 13 | 4 | 4 |  |
| licorice root | 17 | 6 | 4 | 2 | 2 |  |
| mustard | 1 | 1 | 0 | 1 | 1 |  |
| nutmeg | 2 | 1 | 1 | 1 | 0 |  |
| turmeric | 39 | 18 | 9 | 3 | 3 |  |
| vanilla | 5 | 0 | 0 | 1 | 1 |  |
| peppers (all) | 10 | 3 | 4 | 0 | 0 |  |
| cayenne | 5 | 1 | 2 | 0 | 0 |  |
| chili pepper | 4 | 1 | 1 | 0 | 0 |  |
| red pepper | 1 | 1 | 1 | 0 | 0 |  |
| floral (all) | 197 | 81 | 12 | 42 | 6 |  |
| cactus flower | 2 | 0 | 0 | 0 | 0 |  |
| calendula | 14 | 7 | 1 | 3 | 1 |  |
| California poppy | 2 | 0 | 0 | 0 | 0 |  |
| camellia | 2 | 2 | 1 | 1 | 0 |  |
| cape lilac | 1 | 1 | 0 | 0 | 0 |  |
| cherry blossom | 1 | 1 | 0 | 0 | 0 |  |
| chrysanthemum | 1 | 1 | 0 | 0 | 2 |  |
| cornflower | 2 | 1 | 0 | 0 | 0 |  |
| crocus | 1 | 0 | 0 | 0 | 0 |  |
| edelweiss | 6 | 1 | 0 | 2 | 0 |  |
| elderflower | 4 | 0 | 0 | 1 | 0 |  |
| four o’ clock | 1 | 0 | 0 | 0 | 0 |  |
| gardenia | 5 | 1 | 0 | 1 | 0 |  |
| geranium | 10 | 3 | 1 | 1 | 0 |  |
| hibiscus | 8 | 4 | 0 | 3 | 0 |  |
| honeysuckle | 1 | 0 | 1 | 0 | 0 |  |
| jasmine | 9 | 1 | 1 | 2 | 0 |  |
| lavender | 49 | 22 | 2 | 10 | 0 |  |
| lotus | 4 | 4 | 0 | 2 | 0 |  |
| lupine | 1 | 0 | 0 | 0 | 0 |  |
| magnolia | 3 | 2 | 1 | 1 | 1 |  |
| marigold | 5 | 5 | 0 | 2 | 1 |  |
| manuka | 2 | 0 | 0 | 0 | 0 |  |
| Mexican poppy | 2 | 0 | 0 | 0 | 0 |  |
| mimosa flower | 1 | 1 | 0 | 1 | 0 |  |
| nasturtium | 1 | 0 | 0 | 0 | 0 |  |
| neroli | 8 | 2 | 0 | 0 | 0 |  |
| orchid | 1 | 0 | 0 | 0 | 0 |  |
| pansy | 1 | 1 | 0 | 1 | 0 |  |
| passionflower | 6 | 4 | 1 | 0 | 0 |  |
| patchouli | 4 | 2 | 2 | 1 | 1 |  |
| peony | 1 | 0 | 0 | 0 | 0 |  |
| rose | 29 | 10 | 1 | 9 | 0 |  |
| salvia | 1 | 1 | 0 | 0 | 0 |  |
| tansy | 2 | 2 | 0 | 2 | 0 |  |
| tuberose | 1 | 0 | 0 | 0 | 0 |  |
| violet | 2 | 0 | 0 | 0 | 0 |  |
| ylang ylang | 3 | 2 | 0 | 0 | 0 |  |
| herbs – other (all) | 326 | 181 | 49 | 60 | 42 |  |
| arnica | 6 | 5 | 0 | 0 | 0 |  |
| ashitaba | 1 | 0 | 0 | 0 | 0 |  |
| ashwaganda | 25 | 18 | 3 | 7 | 1 |  |
| astragalus | 10 | 7 | 2 | 1 | 4 |  |
| bacopa | 4 | 4 | 1 | 2 | 0 |  |
| bakuchiol | 7 | 3 | 0 | 1 | 0 |  |
| bhringraj | 1 | 0 | 0 | 0 | 0 |  |
| blackberry leaf | 1 | 0 | 0 | 0 | 0 |  |
| boswellia | 2 | 1 | 0 | 0 | 0 |  |
| burdock | 6 | 4 | 4 | 1 | 2 |  |
| butterbur | 2 | 2 | 0 | 0 | 0 |  |
| cassia | 1 | 1 | 0 | 0 | 0 |  |
| catnip | 1 | 0 | 0 | 0 | 0 |  |
| cat's claw | 1 | 1 | 0 | 0 | 1 |  |
| chamomile | 32 | 13 | 3 | 4 | 1 |  |
| chickweed | 1 | 1 | 0 | 0 | 0 |  |
| comfrey | 6 | 4 | 0 | 3 | 0 |  |
| damiana leaf | 2 | 0 | 0 | 0 | 0 |  |
| dandelion | 8 | 3 | 2 | 1 | 1 |  |
| dang gui | 1 | 0 | 0 | 0 | 0 |  |
| duckweed | 1 | 0 | 1 | 0 | 0 |  |
| echinacea | 7 | 6 | 1 | 1 | 2 |  |
| eleuthero | 1 | 0 | 1 | 0 | 0 |  |
| eucommia | 1 | 1 | 0 | 0 | 1 |  |
| feverfew | 4 | 3 | 1 | 0 | 1 |  |
| garcinia cambogia | 3 | 2 | 3 | 0 | 0 |  |
| gingko | 5 | 4 | 0 | 2 | 0 |  |
| ginseng | 11 | 6 | 2 | 2 | 2 |  |
| goldthread | 1 | 0 | 0 | 0 | 0 |  |
| gotu kola | 4 | 1 | 1 | 1 | 1 |  |
| guarana | 3 | 3 | 0 | 1 | 1 |  |
| heal-all | 1 | 1 | 0 | 0 | 1 |  |
| horny goat weed | 1 | 1 | 0 | 0 | 1 |  |
| horsetail | 7 | 3 | 0 | 4 | 1 |  |
| hydrangea leaf | 1 | 1 | 0 | 0 | 0 |  |
| indigo | 1 | 1 | 0 | 0 | 0 |  |
| Irish moss | 2 | 1 | 0 | 0 | 0 |  |
| isatis (woad) | 3 | 2 | 2 | 0 | 2 |  |
| Japanese knotweed | 3 | 1 | 0 | 2 | 2 |  |
| longan | 1 | 1 | 0 | 0 | 1 |  |
| maca | 5 | 3 | 1 | 0 | 2 |  |
| maidenhair | 2 | 1 | 0 | 0 | 0 |  |
| marshmallow root | 10 | 3 | 0 | 3 | 0 |  |
| meadowfoam | 12 | 4 | 0 | 5 | 0 |  |
| milk thistle | 5 | 3 | 2 | 0 | 1 |  |
| moringa | 7 | 4 | 4 | 0 | 0 |  |
| motherwort | 1 | 0 | 0 | 0 | 0 |  |
| mullein | 1 | 1 | 0 | 0 | 0 |  |
| myrtle | 4 | 2 | 0 | 1 | 0 |  |
| neem leaf | 5 | 1 | 0 | 1 | 0 |  |
| nettle | 8 | 6 | 2 | 0 | 2 |  |
| noni | 2 | 0 | 0 | 1 | 0 |  |
| palo santo | 3 | 2 | 0 | 1 | 0 |  |
| plantain | 3 | 2 | 1 | 1 | 0 |  |
| quandong | 1 | 0 | 0 | 0 | 0 |  |
| raspberry leaf | 2 | 1 | 0 | 1 | 0 |  |
| rauwolfia | 2 | 1 | 0 | 0 | 1 |  |
| ravintsara | 2 | 2 | 0 | 0 | 0 |  |
| red clover | 2 | 1 | 2 | 0 | 1 |  |
| rhodiola | 11 | 8 | 2 | 1 | 2 |  |
| rooibos | 2 | 0 | 1 | 0 | 0 |  |
| rose of Jericho | 1 | 1 | 0 | 1 | 0 |  |
| saint John's wort | 2 | 1 | 0 | 0 | 0 |  |
| saw palmetto | 3 | 2 | 0 | 2 | 2 |  |
| schisandra | 5 | 4 | 2 | 0 | 2 |  |
| senna | 3 | 2 | 1 | 0 | 0 |  |
| shatavari | 2 | 2 | 1 | 0 | 1 |  |
| shepherd’s purse | 1 | 1 | 0 | 0 | 0 |  |
| skullcap | 5 | 0 | 0 | 0 | 0 |  |
| Solomon’s seal | 4 | 0 | 1 | 0 | 0 |  |
| speedwell | 2 | 0 | 0 | 0 | 0 |  |
| sweetgrass | 1 | 1 | 0 | 0 | 0 |  |
| thistle | 1 | 1 | 0 | 1 | 0 |  |
| valerian | 7 | 3 | 2 | 0 | 0 |  |
| witch hazel | 11 | 3 | 0 | 3 | 0 |  |
| wormwood | 1 | 0 | 0 | 0 | 0 |  |
| yarrow | 3 | 3 | 0 | 0 | 0 |  |
| yaupon | 1 | 1 | 0 | 0 | 0 |  |
| yerba santa | 1 | 1 | 0 | 0 | 0 |  |
| yohimbe | 3 | 1 | 0 | 0 | 0 |  |
| yerba mate | 2 | 2 | 0 | 1 | 0 |  |
| mushrooms (all) | 36 | 26 | 6 | 4 | 4 |  |
| chaga mushrooms | 5 | 4 | 0 | 1 | 0 |  |
| cordyceps | 8 | 8 | 3 | 0 | 1 |  |
| lion's mane | 9 | 5 | 1 | 2 | 1 |  |
| reishi mushroom | 10 | 7 | 2 | 1 | 2 |  |
| tremella mushroom | 1 | 0 | 0 | 0 | 0 |  |
| maitake mushroom | 2 | 1 | 0 | 0 | 0 |  |
| shitake mushroom | 1 | 1 | 0 | 0 | 0 |  |
| oils/seeds (all) | 160 | 61 | 10 | 53 | 7 |  |
| argan | 23 | 6 | 0 | 17 | 5 |  |
| castor seed oil | 8 | 2 | 0 | 8 | 0 |  |
| CBD | 42 | 15 | 0 | 1 | 0 |  |
| chia | 14 | 6 | 5 | 3 | 1 |  |
| evening primrose | 4 | 1 | 0 | 0 | 0 |  |
| flaxseed | 11 | 7 | 5 | 4 | 1 |  |
| hemp seed | 23 | 10 | 0 | 7 | 0 |  |
| jojoba | 31 | 13 | 0 | 12 | 0 |  |
| palm/palm oil | 4 | 1 | 0 | 1 | 0 |  |
| seaweeds (all) | 35 | 16 | 4 | 13 | 1 |  |
| bladderwrack | 5 | 3 | 1 | 3 | 0 |  |
| sea buckthorn | 8 | 6 | 0 | 3 | 0 |  |
| sea fennel | 3 | 1 | 0 | 1 | 0 |  |
| sea kelp | 9 | 1 | 0 | 4 | 0 |  |
| sea moss | 1 | 1 | 0 | 0 | 0 |  |
| seaweed | 7 | 3 | 2 | 2 | 1 |  |
| wakame | 2 | 1 | 1 | 0 | 0 |  |
| tea (all) | 58 | 24 | 6 | 9 | 5 |  |
| black tea | 9 | 1 | 0 | 1 | 0 |  |
| green tea | 39 | 16 | 4 | 6 | 3 |  |
| matcha green tea | 8 | 6 | 2 | 1 | 2 |  |
| white tea | 2 | 1 | 0 | 1 | 0 |  |
| other products (all) | 137 | 62 | 14 | 30 | 6 |  |
| agave | 7 | 2 | 0 | 1 | 0 |  |
| aloe | 47 | 22 | 4 | 15 | 2 |  |
| bamboo | 10 | 3 | 0 | 5 | 0 |  |
| baobab | 7 | 3 | 0 | 3 | 0 |  |
| birch | 2 | 2 | 0 | 0 | 0 |  |
| cascara | 1 | 0 | 1 | 0 | 0 |  |
| chlorophyll | 2 | 0 | 0 | 0 | 0 |  |
| coffee | 15 | 9 | 3 | 2 | 0 |  |
| oats | 27 | 13 | 5 | 3 | 3 |  |
| oat straw | 1 | 1 | 0 | 0 | 0 |  |
| psyllium husk | 2 | 0 | 1 | 0 | 0 |  |
| purslane | 4 | 1 | 0 | 0 | 0 |  |
| rosewood | 2 | 1 | 0 | 0 | 0 |  |
| shilajit | 1 | 1 | 0 | 0 | 1 |  |
| willow bark | 9 | 4 | 0 | 1 | 0 |  |
